# New Insights in Prevention and Treatment of Cardiovascular Disease

**DOI:** 10.3390/ijerph19042475

**Published:** 2022-02-21

**Authors:** Domenico Di Raimondo, Gaia Musiari, Giuliana Rizzo, Edoardo Pirera, Salvatore Santo Signorelli

**Affiliations:** 1 Department of Promoting Health, Maternal-Infant, Excellence and Internal and Specialized Medicine (Promise) G. D’Alessandro, Division of Internal Medicine and Stroke Care, University of Palermo, 90100 Palermo, Italy; gaiamusiari@gmail.com (G.M.); giulianarizzo@yahoo.it (G.R.); edoardo.pirera95@gmail.com (E.P.); 2 Medical Angiology Unit, Department of Clinic and Experimental Medicine, University of Catania, 95124 Catania, Italy; ssignore@unict.it

## 1. Introduction

Cardiovascular (CV) disease (CVD) is still a major cause of morbidity and mortality in many countries in Europe although considerable efforts have been made in recent decades to address this disease in an even more “comprehensive” approach [1].

The so-called “traditional” CV risk factors are arterial hypertension, dyslipidemia, diabetes, cigarette smoking, family history of CVD, advanced age (over 65 years of age) and male sex. The presence of one or more of these risk factors predict more than 90% of major cardiovascular events [2]. Of these, the four main modifiable CVD risk factors are dyslipidemia (being low-density lipoprotein (LDL) plasmatic level the main therapeutic target), high blood pressure (BP), cigarette smoking, and diabetes mellitus (DM). Obesity, in view of the dramatic increase in prevalence observed in recent decades and foreseeable in the next and given the significant impact of morbidity resulting from it, should be added to the previous ones [1]. These risk factors are universally perceived as priority targets to achieve the goal of reducing the incidence and prevalence of CVD. Nevertheless, taking into account both health policy interventions and the availability of new and more effective pharmacological therapies, not only do many of these risk factors show no signs of losing ground, their prevalence tends to increase in many countries [1,3]. There is clearly still a great deal to do.

Above the pharmacologic control of risk factors, the importance of firstly promoting a healthy lifestyle should be emphasized; despite the huge amount of evidence available today, this fundamental approach to CV prevention and treatment still needs to be highlighted. A comprehensive intervention that promotes a healthy lifestyle in an individual (abolition of cigarette smoking, regular physical activity, body weight control, adoption of a Mediterranean diet, etc.) would allow a cross-sectional health benefit that would go well beyond CVD improvement and control [4,5,6,7,8,9,10,11,12,13]. A healthy diet, especially the Mediterranean diet, has been demonstrated to significantly influence CV risk, actively contributing to the control of major risk factors such as hypercholesterolemia, high BP, diabetes and obesity [5,8,14].

Indeed, and this must be strongly stated, it is much easier to prescribe a pill than to closely follow a patient assisting him in a path, which is often long and difficult, towards a healthier lifestyle. For this reason, incorrect lifestyle habits still represent the rule, and a healthy lifestyle the exception [1].

The challenge of reducing the risk of developing CVD in a specific patient begins with appropriate assessment of the individual risk, continues with an effective and appropriate patient/doctor communication and interaction and finally depends on the use of tailored (pharmacological and nonpharmacological) interventions which are sufficiently effective to achieve the individual target [1,3,15,16]. The need to explore innovative approaches in the prevention and treatment of CVD is, therefore, justified by the existing difficulty, even today, to adapt the most correct approach to the individual, also in view of the many co-factors that act as “risk modifiers” (ethnicity, psychosocial factors, stress, advanced age, frailty, accessibility of resources, familiarity, heredity, socioeconomic factors, work occupation, environmental pollutants, non-cardiovascular comorbidities, sex-associated factors, etc.).

Notwithstanding what we still do not know about the conditions predisposing to CVD, systemic inflammation (both inflammation resulting from chronic coexisting diseases such as rheumatoid arthritis, psoriasis, active inflammatory bowel disease or ankylosing spondylitis but also chronic low-grade systemic inflammation associated with central obesity and DM) may be a target for both the prevention and treatment of CVD [17,18,19,20,21,22] as demonstrated by the CANTOS [23] study and the COLCOT [24] study. Even in this area, lifestyle interventions can be useful; much evidence has shown that a healthy lifestyle (especially if associated with regular exercise) is also able to grant an anti-inflammatory effect that can be demonstrated with the evaluation of markers of systemic inflammation such as C-reactive protein or pro-inflammatory cytokines [9,10,13,25,26]. 

Therefore, another critical issue to be considered when aiming to prevent and treat CVD in the best way is the close interconnection between CVD and many other chronic non-atherosclerotic diseases. Inside the infectious diseases, for example, it has been demonstrated that an increased risk of CVD independently by known risk factors features various pathologic conditions such as human immunodeficiency virus infection [27,28,29], respiratory tract infections due to multiple different types of virus [30,31], and periodontitis [32,33,34]. 

The same may be observed for many chronic respiratory diseases: COPD, asthma (or the overlapping syndrome between these two conditions, named ACOS), or in subjects suffering by Obstructive Sleep Apnea Syndrome (OSAS). It is now established that these diseases considerably and independently increase the risk of arterial hypertension [35,36,37], CVD, both atrial and ventricular arrhythmias, and heart failure [38,39,40,41,42]. 

Non-alcoholic fatty liver disease (NAFLD) has also been associated by many authors with an increased CV risk independent of traditional risk factors [43]. It is of course possible that inflammation, which in different ways links all the diseases we have just considered, could finally be the lowest common denominator to which the observed excess of CV risk can be allocated but, regardless of the pathophysiology, it remains the clinical finding that requires us to carry out a thorough screening in all these patients to avoid underestimating the real CV risk that affects them.

Another effective intervention strategy in this area is the identification of new serum or urinary biomarkers able to detect at an early stage patients at higher risk of developing CVD, allowing a better attribution of the correct level of risk and thus improving the therapeutic approach and the most appropriate level of follow-up. Biomarkers are analytical tools that define biological parameters. Their main characteristic is that of being able to be measured objectively and to be considered as indicators of biological processes and of response to therapeutic treatments. They assume clinical relevance when they are accurate, reproducible, easy to interpret, highly specific and sensitive for the biological parameter for which they are investigated [44]. In recent decades, many new CV biomarkers have been proposed: polypeptides; hormones and enzymes (natriuretic peptides, copeptin, aldosterone, renin); acute phase proteins and various components of the systemic inflammation cascade (C-reactive protein, interleukins, tumor necrosis factor-α); vascular adhesion molecules (ICAM-1 and VCAM-1); markers of myocardial necrosis (troponin I, myoglobin, creatine phosphokinase); constituents of platelet adhesion to the endothelium and of the coagulation cascade (plasminogen activator inhibitor, D-dimer, factor VII, factor VIII, fibrinogen); markers of lipid balance (HDL-cholesterol, lipoprotein(a) and apolipoprotein J); homocysteine; and uric acid. Only some of these biomarkers are featured by sufficient levels of sensitivity, specificity, reproducibility, low cost and ease of sample collection that have led to their wide and consolidated use in clinical practice, while many others are just occasionally used. However, this is an extremely fertile field of research [45,46,47].

A field of innovation in CVD prevention is also represented by new tools derived from the application of “Information and Communication Technology” in the medical field (smartphones, smartwatches, activity trackers, heart rate monitors, telemeters, etc.). These devices allow the detection and storage of vital parameters and other health information, their remote transmission, analysis and creation of detailed and individualized reports. The field of telemedicine, telemonitoring and use of vital parameters collected by wearable devices for clinical and therapeutic purpose is likely to be a developing area that brings us into a future of tremendous possibilities [48,49,50].

## 2. What Is New in Prevention and Treatment of Cardiovascular Disease

Our Special Issue, in a short period of time, has collected 13 published articles, along with many others evaluated and not considered suitable for publication. This numbers show, given the wide variety of topics covered, how much there is still to research in this area, and how many gaps in knowledge there are to be filled.

The first article we published, in chronological order, by Sharifi-Rad, J. et al. [51], is an excellent review regarding use of pharmacologically active natural compounds (polyphenolic compounds, peptides, oligosaccharides, vitamins, unsaturated fatty acids) as a complementary therapy in cardiovascular disease. It is a very complete and very useful review, and I wholeheartedly invite you to read it.

The following, an excellent review written by Prof. Signorelli’s group [52], discusses a very hot topic, i.e., the most appropriate therapeutic approach for stroke prevention which can objectively improve our clinical practice.

A systematic review and meta-analysis by Saz-Lara, A. et al. [53], aimed to provide a synthesis of the evidence regarding the association of arterial stiffness measured by pulse wave velocity and atherosclerosis measured by carotid intima media thickness with advanced glycation end products assessed by skin autofluorescence. An innovative approach to indirectly estimate vascular subclinical damage.

The fourth and final review we have accepted for publication addresses an extremely topical issue, debating a specific topic related to the current COVID-19 pandemic. In this review, Di Fusco, S.A. et al. [54] summarizes the available data about the effects of lockdown measures, particularly working from home, on cardiovascular risk factors including sedentary lifestyle, unhealthy diet pattern, psychological distress, smoking, alcohol misuse, and cardiometabolic parameters, also suggesting some countermeasures that can attenuate the negative health impact of working from home. Given also the real possibility that our habits will be affected by the pandemic for a long period of time, the resulting changes in cardiovascular risk must be kept in the utmost consideration.

The retrospective survey of the relationship between central serous chorioretinopathy and various types of CVDs with different severity using the Taiwanese National Health Insurance Research Database is the aim of the study by Hsu, H. et al. [55]. The main finding of the study is that central serous chorioretinopathy is correlated with a higher rate of chronic CVD occurrence in the middle-aged male Taiwanese population (HR: 1.391).

Cerebrovascular diseases are also investigated in another original article published in our Special Issue. Magdič, J. et al. [56] assessed the impact of vertebrobasilar artery calcification on the long-term risk for recurrent stroke and vascular events. A total of 448 patients were admitted to the University Hospital of Maribor, Slovenia and followed for a median time of 1505 days. The results suggest that the presence of vertebrobasilar artery calcification in patients with ischemic stroke is a short- and long-term prognostic factor for stroke recurrence and subsequent manifestation of acute vascular disease, proposing an additional tool for quantifying the risk of events.

The impact of climate, weather, and seasonality variables on the incidence of CVDs has always been a fascinating topic. In our Special Issue, Maciejczak, A. et al. [57] investigated the relationship between the occurrence of the Foehn wind, as well as the related environmental variables, and the incidence of cardiac events in the population of southern Poland, a region affected by this type of wind. The frequency of admissions on halny days did not differ significantly from the admissions on the remaining days of the year (*p* = 0.496).

The original article by Prof. Di Raimondo et al. [35] analyzes a part of data collected within the BADA (blood pressure levels, clinical features and markers of subclinical cardiovascular damage of asthma patients) study, aiming to evaluate the prevalence of the cardiovascular comorbidities of asthma and their impact on the clinical outcome of the disease. The authors’ results support the finding that hypertension is highly prevalent in asthma in Italy (75% of the overall sample enrolled) and that the systematic use of ambulatory blood pressure monitoring (ABPM) in chronic asthmatic population could allow us to detect a considerable number of unrecognized hypertensives.

Another elegant study investigating high blood pressure has been developed by Li, F. et al. [58]. The authors explored the trajectories of blood pressure among the youth and middle-aged non-hypertensive Chinese population and CV risk, finding statistically significant associations mainly between systolic blood pressure trajectories and stroke and myocardial infarction. Determinants of pre-hypertension in 2225 normotensive and pre-hypertensive healthy young adults enrolled in the Korean National Health and Nutrition Examination Survey 2018 were investigated by Jang I [59]. Factors related to pre-hypertension in young adults were age, smoking, waist circumference, diabetes, anemia, cholesterol levels including HDL cholesterol, and uric acid levels. The findings of these two studies, as well as others like them, suggest the need for more rigorous control of CV risk factors in clinical practice.

Another popular area of CV research is related to gender differences. Additionally, this topic has also been addressed in our Special Issue by Huang, J.-H. et al. [60] who explore the role of gender in the relation of high-sensitivity C-reactive protein, white blood cell count, and serum uric acid to the risk of future CVD events in 404 Taiwanese workers using the Framingham Risk Score to estimate the risk. With respect to CVD prevention, the white blood cell count (but not the high-sensitivity C-reactive protein) can be used to monitor the risk for all workers. Due to a gender difference shown in the relationship between serum uric acid and the Framingham Risk Score, serum uric acid can appears to be useful to estimate the risk of future CVD events in male Taiwanese workers only.

New insights about the predictors of myocardial infarction, assessed using the Center for Disease (CDC) Control Behavioral Risk Factor Surveillance System (BRFSS) survey for the year 2019 are provided in the paper by Dolezel et al. [61]. Age, gender, marital status, veteran status, income, home ownership, employment status, and education level were important demographic and socioeconomic predictors.

Finally, Kim, K. et al. [62] investigated the relationship between serum uric acid and CVD risk in non-alcoholic fatty liver disease (NAFLD) in a Korean population, using data from the 2016–2018 Korean National Health and Nutrition Examination Survey. Ten-year CVD risk was estimated using an integer-based Framingham risk score. Compared with the lowest uric acid quartile group, the highest quartile group also showed a significantly higher risk of having CVD (OR 2.76; 95% CI 2.34–3.25) after adjustment (OR 4.01; 95% CI 3.37–4.78), suggesting that serum uric acid level is independently associated with CV risk in NAFLD.

## 3. Conclusions

New advances in the prevention, diagnosis, and treatment of main cardiovascular disease as well as CV risk factors are required in order to improve the ability to (1) identify patients at higher risk early on; (2) identify early markers of subclinical cardiovascular disease; (3) improve treatment algorithms for the different clinical syndromes; (4) assess residual risk and follow-up optimization; (5) prevent recurrences. This is the challenge of the forthcoming decades, and the clinicians of the future must be ready to meet it.
